# Large-scale rheological and tectonic effects of localized garnet breakdown: a case from the Pannonian Basin

**DOI:** 10.1038/s41598-026-43790-4

**Published:** 2026-04-18

**Authors:** Kristóf Porkoláb, Kálmán Török, Tamás Spránitz, István János Kovács, Eszter Békési, Márta Berkesi

**Affiliations:** 1 HUN-REN Institute of Earth Physics and Space Science, Sopron, Hungary; 2 MTA-HUN-REN FI FluidsByDepth Momentum Research Group, Sopron, Hungary; 3 Department of Geology and Laboratories, SARA (Supervisory Authority for Regulatory Affairs) Geological Survey, Budapest, Hungary

## Abstract

The accurate representation of continental lower crust rheology is critical for modelling plate tectonic processes. However, limited observations and highly heterogenous composition make it difficult to describe its large-scale behaviour. Here we aim to link local heterogeneities to large-scale behaviour through a case study from the Pannonian Basin. Available samples of the lower crust are dominantly strong and dry, garnet-rich mafic granulites. In contrast, inferences from large-scale tectonics suggest a weak rheology for the lower crust. We combine observations and numerical models to show that networks of weak zones related to fluid percolation and garnet breakdown surround the strong domains. Strain localization in the weak zones significantly decreases overall long-term stress magnitudes. Consequently, lower crust domains that mainly consist of strong lithologies may still behave as weak layers in the lithosphere due to reaction-induced long-term weakening. Strength increases significantly when weak zones are scarce or discontinuous.

**Supplementary Information:**

The online version contains supplementary material available at 10.1038/s41598-026-43790-4.

## Introduction

The crucial effects of lithosphere rheology on plate tectonic processes have long been demonstrated for all stages of the Wilson-cycle^1,2^, such as rifting^3–6^, subduction^7–9^, or mountain building^10,11^. The strength of the lithosphere depends on its structure and rheological properties; most importantly the mineralogical composition, pressure, temperature, strain rate, fluid content, and fluid properties. Rheological models of continental plates typically assume a three-layer model consisting of a felsic upper crust, a felsic or mafic lower crust, and an olivine-dominated mantle^12–14^. In the context of such models, assessing the large-scale rheology of the lower crust has been a challenge due to its heterogenous composition and very limited available direct observations. Rheological representation of the lower crust may rely on large-scale geophysics-based composition models^15–17^, which indicate a generally mafic (mafic granulite) composition for the global lower crust^17^. Further key sources of information are xenolith samples brought up by volcanism^18–20^, and outcrops of exhumed, former lower crust bodies^21–23^.

**Fig. 1:**
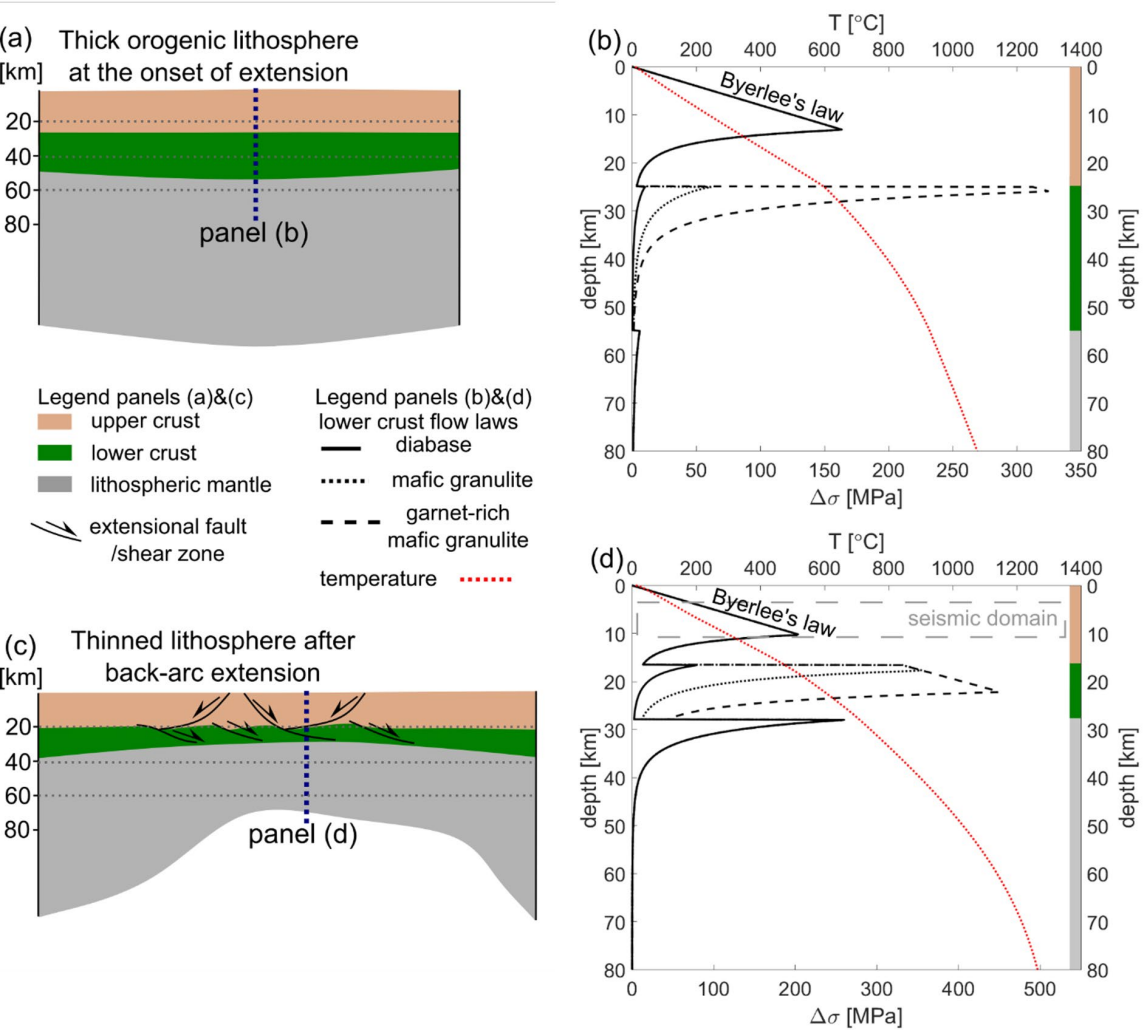
Structure and strength of the lithosphere before and after the extension of an overthickened orogenic area, mimicking the Pannonian Basin case. **a** Schematic section of a thickened orogenic lithosphere at the onset of extension. The thickness of lithosphere layers are based on present-day maximum values in the Alps^29^ and on estimations from lower crust xenoliths found in the Pannonian Basin^30^. **b** Strength (yield stress, Δ**σ**) of the thick orogenic lithosphere. Brittle yield stress^31^ is calculated for the onset of extension (stress regime coefficient in Byerlee’s law is β = 0.75). Flow laws of the lower crust are based on^27^ (diabase) and^28^ (mafic granulite), respectively. Garnet-rich mafic granulite flow law is defined as a 50–50% mixture of garnet-free mafic granulite^32^ and garnet^33^, using the aggregate law of^34^. Upper crust is based on^35^ (granite), mantle is based on^27^ (olivine). Temperature gradient is defined using the numerical modelling approach of^36^, assuming steady-state conductive heat transfer and a crustal and lithospheric thickness of 55 km and 130 km, respectively. (**c**) Schematic section of the post-extension lithosphere. The depth of lithosphere layers within the basin (top and bottom of lower crust is 18 and 28 km, lithosphere-asthenosphere boundary is 75 km) are based on^37,38^. **d** Strength of the extended lithosphere. Flow laws are the same as in panel (**b**). Brittle yield is calculated for the end of extension (β = 1.2 for a strike-slip regime). Temperature profile is based on^36^, at the location of the BBHVF (Fig. 2a). Grey dashed polygon indicates the characteristic depth range of seismicity in the Pannonian Basin^39–41^.

Numerous field studies of exhumed lower crust outcrops indicate a heterogenous mineralogical composition (and hence heterogenous rheology) from cm to km scale, which may arise due to fluid-rock interactions and/or metamorphic reactions within an originally more homogenous lower crust body^22,24–26^. These cases highlight the difficulty of describing the mechanical behavior of the heterogenous lower crust with a single flow law in large-scale geodynamic models. Choosing a relatively weak (e.g. Maryland diabase^27^) or strong (e.g. mafic granulite^28^) material for the lower crust (see strength differences in Fig. 1b,d) yields dramatically different outcomes for both rifting and subduction/collision processes^3,10^. Therefore, understanding and quantifying the effect of rheological heterogeneities on the overall mechanical behavior of the lower crust is critical for the modelling of plate tectonic processes.

**Fig. 2:**
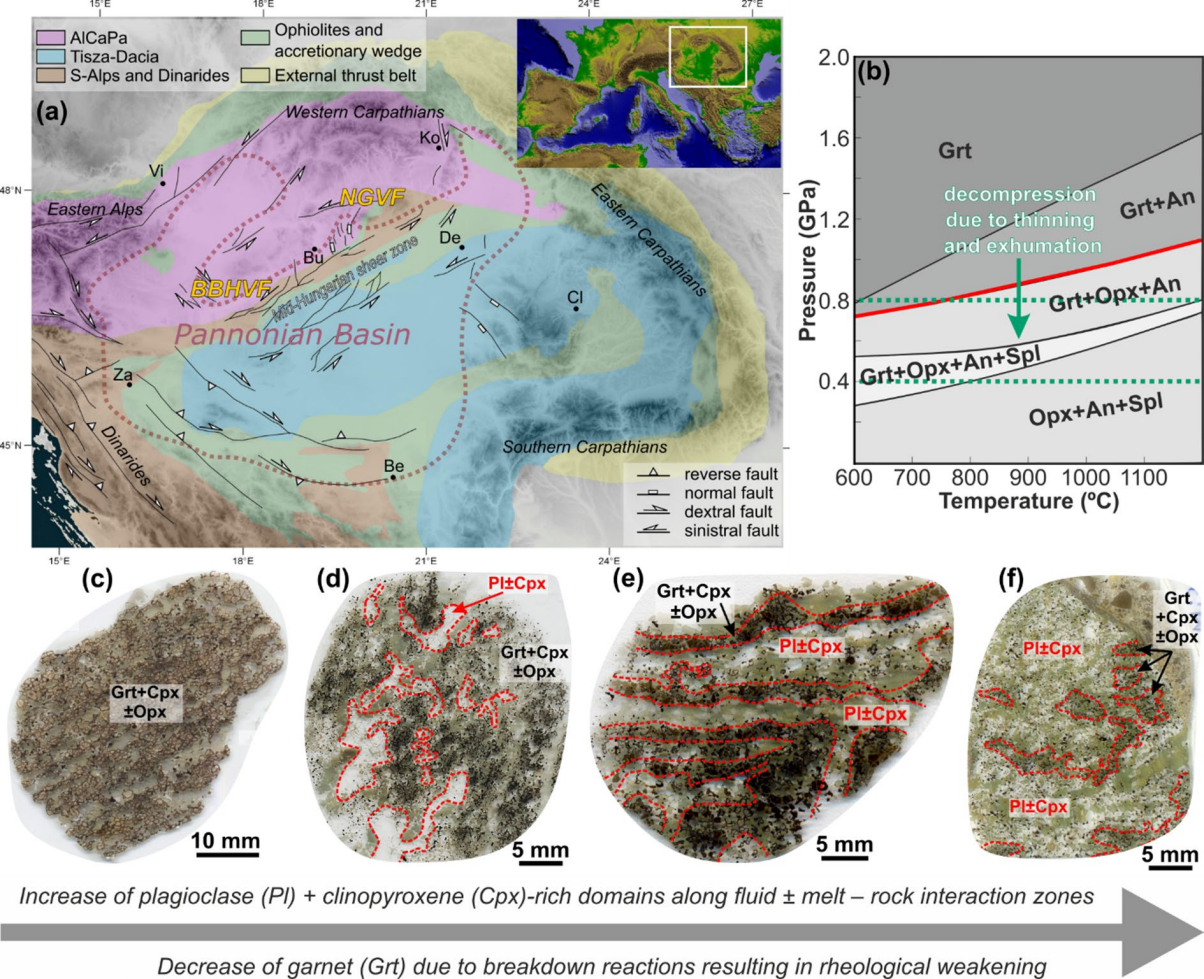
**a** Map of the Circum-Pannonian region modified after^42^ showing main geographic and tectonic units. BBHVF: Bakony-Balaton Highland Volcanic Field; NGVF: Nógrád-Gömör Volcanic Field; Vi: Vienna, Ko: Kosice, Bu: Budapest, Za: Zagreb, De: Debrecen, Cl: Cluj-Napoca, Be: Belgrade. **b** Pseudosection showing phase assemblages in the CFMAS system using Perple_X^43^. The phase diagram was calculated for the same garnet composition and parameters as described in^44^. Red line denotes P–T conditions where garnet breakdown initiates when pressure decreases (green arrow), which occurred during the thinning and exhumation of the lower crust in the Pannonian Basin^30,44,45^. Dotted green lines indicate the approximate pressure limits of the present-day lower crust, assuming that pressure is lithostatic. **c–f** Photomicrographs showing typical rock textures and mineral assemblages of lower crustal granulite xenoliths on scanned images of thick sections from the BBHVF (Pannonian Basin). The extent of garnet breakdown along fluid/melt interaction zones increases from left (**c**) to right (**f**). All figure panels were edited with the software Inkscape (https://inkscape.org/), version 1.3.

Here we focus on the lower crust beneath the Pannonian Basin (Central Europe), which shows an apparent contradiction between its large-scale mechanical behavior and observed lithological composition. The large-scale deformation history of the Pannonian Basin offers multiple inferences for the general weakness of the lower crust. First, the wide rift style of Miocene back-arc extension resulting in widepread extension and the formation of detachment systems accomodating significant crustal exhumation^46–50^ would not have been possible without an easily flowing, weak lower crust^3,5,6,47,51^. Second, the pattern of large-scale contractional bending of the lithosphere during Late Miocene-to present inversion of the Pannonian Basin^52,53^ requires a relatively thin brittle layer (upper crust) underlain by a weak ductile lower crust^53,54^. Third, the depth distribution of seismicity in the Pannonin Basin shows a lack of lower crustal earthquakes (Fig. 1d), indicating that the upper crust is the only brittle layer in the lithosphere^39–41^.

However, lithological observations paint a different picture. The lower crust of the Pannonian Basin was sampled by Mio-Pleistocene monogenetic, alkali basalt volcanism^55^, bringing up numerous lower crustal xenoliths that exhibit a dominantly very strong, garnet-rich mafic granulite compositionin in the Bakony-Balaton Highland (BBHVF), and the Nógrád-Gömör (NGVF) volcanic fields (Fig. 2a)^20,30,56,57^. The flow stress of garnet-rich mafic granulites far exceeds that of any commonly used lower crust flow laws (such as Maryland diabase^27^), and would predict high differential stresses and even brittle conditions for significant parts of the pre-extension as well as present-day lower crust (Fig. 1b,d), not matching the inferences from large-scale tectonics outlined above.

We aim to resolve this apparent contradiction by assessing the mechanical role of reaction-induced weakening in the lower crust throughout the evolution of the Pannonian Basin. We show based on lower crust xenolith samples from the BBHVF that relatively weak reaction zones linked to fluid/melt infiltration surround the strong and exceptionally dry garnet-rich mafic granulite bodies in the Pannonian lower crust. The H_2_O content of 15 lower crustal xenoliths is quantified by new micro Fourier-transform infrared spectrometry (micro-FTIR) data. We then present visco-elastic numerical models to quantify the mechanical behavior of strong non-reacted lower crust domains penetrated by retrogressed weak zones. Model results are compared to flow laws of typical lower crust lithologies, allowing to bridge the gap between the rheological description of heterogenous lower crust domains and that of homogenous large-scale models.

### Geological setting

The Pannonian Basin is a Miocene extensional basin formed in the back-arc of the Carpathian subduction zone^46,58,59^ (Fig. 2a). Initial extension occurred at overthickened orogenic areas in the Adria-Europe collision zone, accompanied by lateral extrusion from the Alps towards the Pannonian Basin^60^. Lateral extrusion resulted in the juxtaposition of the Adria-derived AlCaPa (Alps-Carpathians-Pannonia) and the Europe-derived Tisza-Dacia nappe stacks (Fig. 2a), former undergoing counterclockwise, latter clockwise rotation^61–63^. These units constitute the pre-Cenozoic basement of the Pannonian Basin, now covered by several kilometers of sediments in the sub-basins. The locking of the Carpathian subduction zone in the Late Miocene caused a shift from extension in the basin towards strike-slip and compression regime^58^, leading to the so-called “neotectonic” inversion in the Pannonian Basin, which is still active^41,64–66^.

The composition of the AlCaPa lower crust in the Pannonian Basin can be directly studied by means of xenoliths brought up by Pliocene basalts in the BBHVF and the NGVF (Fig. 2a). Garnet-bearing mafic granulite is the predominant lower crustal rock-type in the BBHVF while it is the sole rock type in the NGVF^20,56,57^. Some of the xenolith localities of the BBHVF also exhibit felsic granulites that are also rich in garnet^30^. Mafic granulites underwent partial melting, which enriched the rocks in garnet and pyroxene. Peak temperature and pressure recorded in the lower crustal granulites is 800–1050 °C and 1–1.6 GPa^20,57^, which are much higher than the values estimated for the present-day lower crust affected by Miocene thinning (Fig. 1c), exhumation, and post-extension cooling^30,36,37^. Decreasing pressure in the lower crust during Miocene extension resulted in widespread garnet breakdown reactions in the granulites^30,44,45^.

## Results

### Composition of lower crust xenoliths

**Fig. 3:**
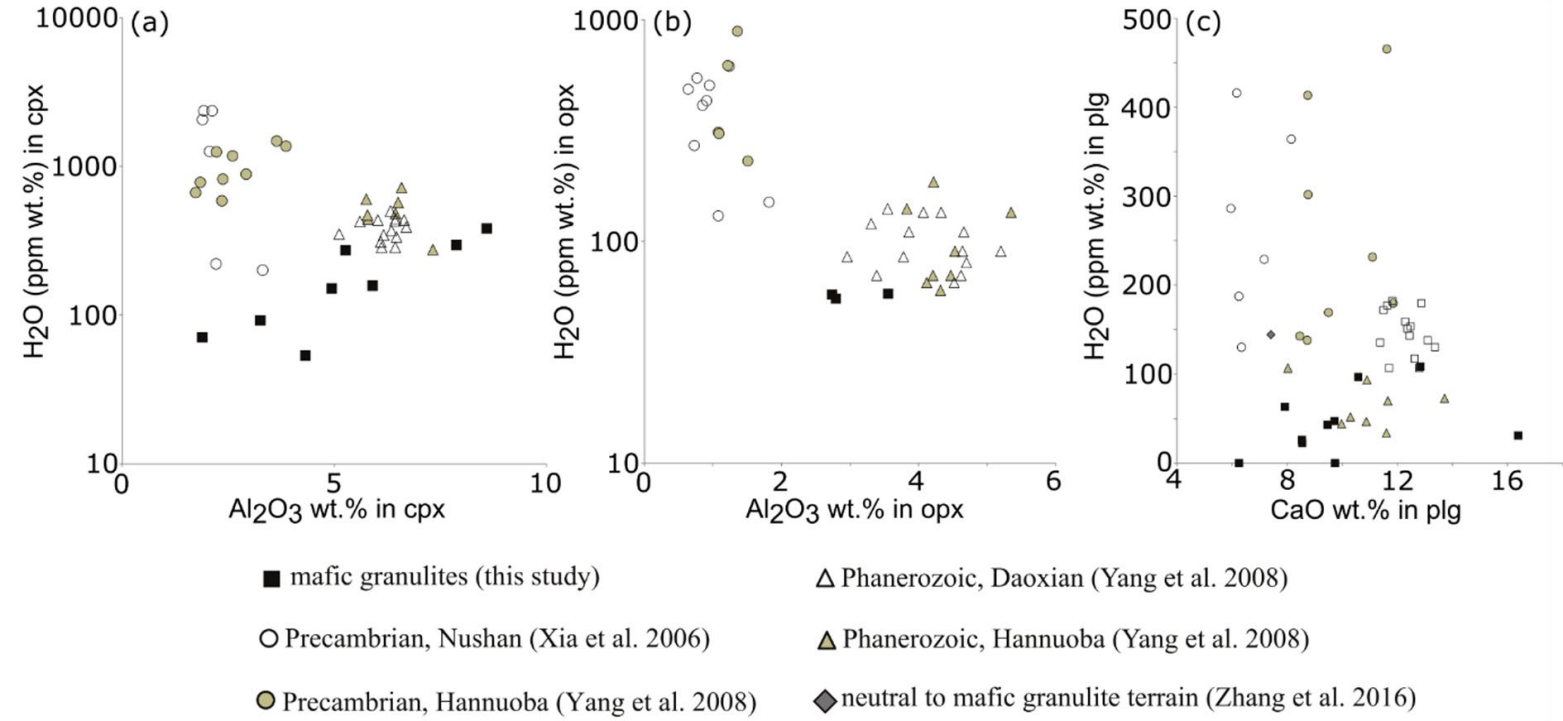
H_2_O contents of lower crust xenoliths from the Bakony-Balaton Highland (for all measurements see Table S1). Comparison to Precambrian and Phanerozoic granulite xenolith data from the North China Craton^69,70^ and a metamorphic granulite terrain in the Central Asian Orogenic Belt^71^ highlights the dry nature of the Pannonian lower crust. **a** H_2_O versus Al_2_O_3_ content in clinopyroxenes. **b** H_2_O versus Al_2_O_3_ content in orthopyroxenes. **c** H_2_O versus CaO content in plagioclase. Garnet in these samples is generally so dry that the H_2_O content is below detection limit, preventing the plotting of these results.

Mafic granulite xenoliths show abundant evidence for fluid/melt migration and reactions with the wall rock, possibly focussed along pre-existing permeable fracture zones^67^. Such fluid-rock interaction zones produced clinopyroxene-plagioclase-rich and/or amphibole-rich zones, which are free of garnets (Fig. 2), suggesting that fluid presence—besides pressure decrease—is a key factor in garnet breakdown reactions^44,45,57^. The degree of retrogression and hence the proportion of garnet-free zones in the lower crustal xenoliths is highly variable. Parts of the lower crust shows absolutely no sign of retrograde reactions; the lithology consists entirely of garnet and pyroxene (Fig. 2c), representing a rheologically extremely strong part of the lower crust (Fig. 1d). Numerous samples exhibit minor-moderate degree of garnet breakdown, with well-defined zones of the original garnet-rich and the newly formed garnet-free, plagioclase-and pyroxene-rich lithologies (Fig. 2d,e). At the other end of the spectrum, the almost complete breakdown of the original garnet-rich lithology can also be observed (Fig. 2f), resulting in a rock characterized by relatively weak rheology. Furthermore, it has been demonstrated that the minerals of the alteration zones/veins are enriched in water and contain water-bearing melt and fluid inclusions, in contrast to the dry garnet-rich domains of the wall rock^68^. The water content of the local alteration zones can reach concentrations 5 times higher than that of the main garnet-rich lithology (278 ± 83 vs. 55 ± 17 ppm^68^). The exceptional dryness of garnet-rich domains might be related to multiple generations of fluid and melt extraction from the lower crust^30,57,68^. Therefore, alteration zones are rheologically weaker due to two factors: (1) mineralogy (garnets replaced by weaker plagioclase and pyroxene); and (2) higher water content. The very low water content of the garnet-rich mafic granulite domains, is furthermore demonstrated below by new micro-FTIR spectrometry data (Fig. 3, Table S1).

### Water content of lower crust xenoliths

We measured the water content of 11 mafic and 4 felsic (metapelitic), garnet-bearing granulite xenoliths from the BBHVF with micro-FTIR spectrometry (for all water content data, sample locations, and IR spectra plots see Tables S1, S2, and Fig. S1, respectively). Garnet typically does not display any significant absorption band on the FTIR spectra (Fig. S1), and apart from four samples have no detectable water (Table S1). In the four samples that contain detectable water, however, the water-related absorption bands (i.e. those at 3270 and 3400 cm^−1^) are only present if fluid and/or melt inclusions are visible in the analysed area, otherwise garnets are extremely dry (water content is below the limit of detection). Water contents measured in garnets reported from numerous locations worldwide indicate significantly higher concentrations of water (generally a few hundred to a few thousand ppm) in most cases^69–72^. Water content in orthopyroxene and plagioclase crystals in our samples is generally below 100 ppm, also indicating very dry conditions with respect to literature data^72^ (Fig. 3b,c). Clinopyroxene is generally more water rich containing more than 100 ppm water; however, it is still dryer than most of the literature examples (Fig. 3a). Clinopyroxene water content shows a correlation with Al_2_O_3_ content (Fig. 3a), which may indicate coupled Al^3+^ + H^+^ substitution for Si^4+^.

### Numerical model

**Fig. 4:**
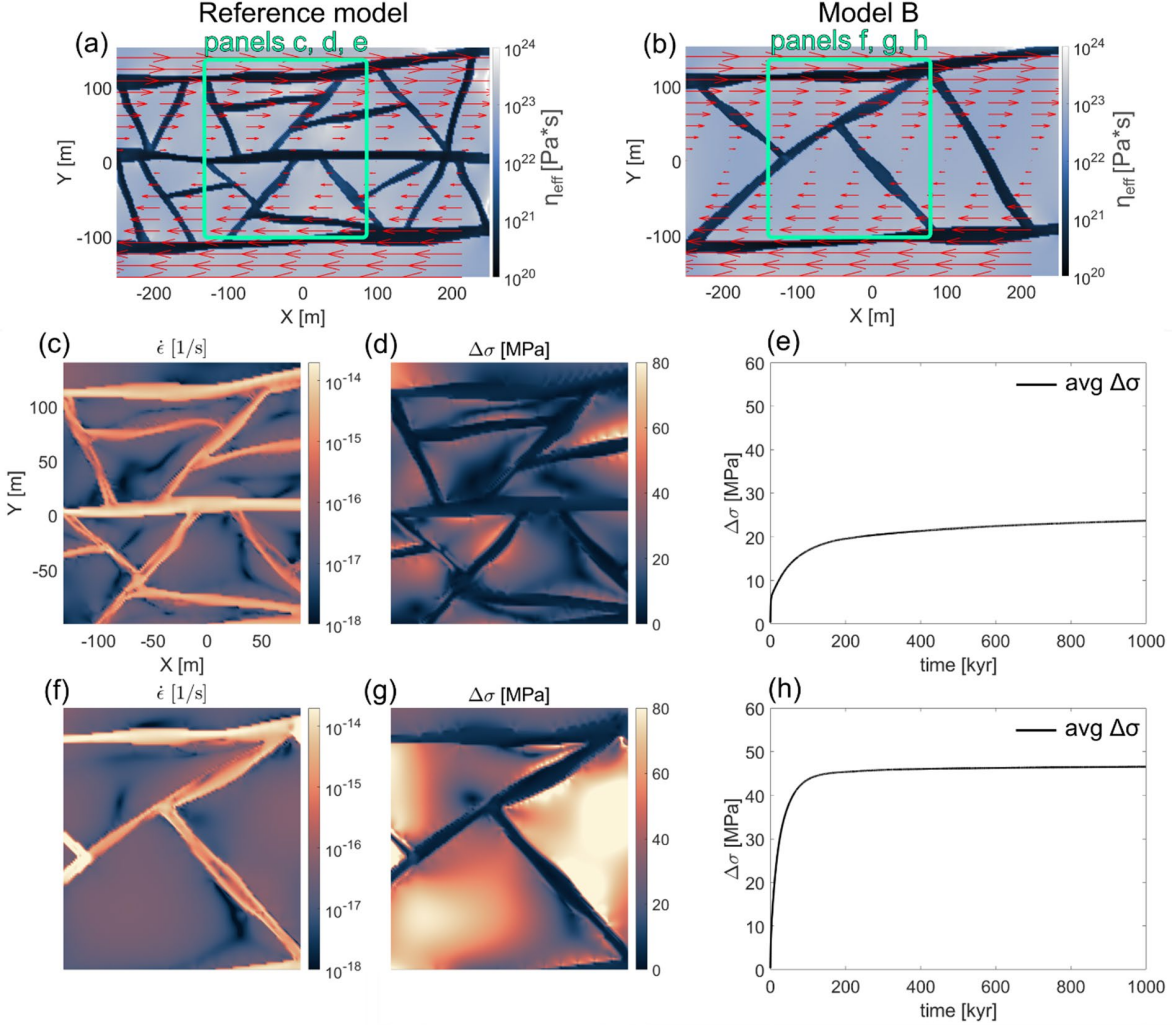
Initial conditions and evolution of the reference and model B setups. The difference between the two setups is only the geometry of the weak zone network. **a** Effective viscosity field (η_*eff*_) after the first time step (50 years) and the initial imposed velocity field (red arrows) of the reference model, where retrogressed weak zones constitute a dense, continuous network (27% of the model area) and surround the dry, garnet-rich mafic granulite bodies. Velocity gradients define simple shearing with a rate of 10^–15^ s^−1^. Boundary velocities are fixed. Effective viscosities are based on moderately wet mafic granulite^76^ for the weak zones, while a 50–50% mixture of garnet-free mafic granulite^32^ and garnet^33^ (using the aggregate law of^34^) is adopted for the dry, garnet-rich mafic granulites. **b** Effective viscosity field after the first time step and the initial imposed velocity field of model B, where weak zones are scarcer (17% of the model area). **c** Strain rate ($\dot{{\boldsymbol{\varepsilon}}}$) in the reference model after 70 kyr. **d** Differential stress ($\Delta$**σ**) in the reference model after 70 kyr. **e** Time evolution of average differential stress in the reference model. Average differential stress is tracked within the domain of panels c, d, f, and g, to avoid any boundary effects at the edges of the model. **f** Strain rate in model B after 70 kyr. (**g**) Differential stress in model B after 70 kyr. **h** Time evolution of average differential stress in model B.

To investigate the deformation pattern and characteristic stress levels of a heterogeneous lower crust body subjected to tectonic deformation, we designed a series of 2D visco-elastic numerical simulations (see *Methods* section for the details of the numerical model, and Figs. S2–S6 for parameter tests). Model geometry mimics the first-order structural and compositional features of the mafic Pannonian lower crust (Fig. 2). The model is a 0.5 km × 0.3 km rectangle consisting of garnet-rich mafic granulite blocks that are surrounded by a relatively thin network of moderately wet, garnet-free mafic granulite, assumed to be produced by fluid/melt-assisted retrograde reactions (as observed in the xenoliths, Fig. 2). Since we only have xenolith-based, cm-scale observations from the Pannonian lower crust, we also rely on observations from analogous exhumed lower crust bodies for geometrical patterns and scales. It has been shown that the pattern of fluid-induced retrogression is scale-independent, meaning that cm-scale patterns of weakening are likely to be present also on the km-scale^25^. Furthermore, it has been demonstrated that fluid-induced weakening along fracture/fault zones within an initially strong lower crust may develop network-like weak zones^73^, matching field observations^74,75^. The pattern of the weak zones in our model is on purpose not symmetric, and its boundaries are not straight, mimicking a realistic geometry (Figs. 2c–f and 4). The thickness of individual weak zones ranges from a few meters to few tens of meters, which could correspond to small and moderate fault/shear zones. We use a power-law effective viscosity model: garnet-free and wet weak zones are defined by the flow law of moderately wet, weak mafic granulite^76^, while garnet-rich, dry domains are defined by garnet-free mafic granulite^32^ and garnet^33^ flow laws in a 50–50% mixture (using the aggregate law of^34^). These approximations yield an effective viscosity contrast of ~ 2 orders of magnitude between weak and strong domains for a homogenous strain rate of 10^–15^ s^−1^ (Eq. 9). This contrast is further enhanced by strain localization in the weak zone network after deformation initiates (see the 3–4 orders of magnitude contrast after the first model time step, Fig. 4a,b).

The model domain is subjected to simple shearing with a strain rate of 10^–15^ s^−1^ (Fig. 4a,b), representing a moderately deforming area. This results in visco-elastic deformation and the buildup of differential stress (Fig. 4). The rate of stress buildup is decreasing continuously—due to decreasing elastic strain rates—and reaches a characteristic long-term level after ~ 100 kyr (Fig. 4e,h). Garnet-free weak zones accommodate most of the deformation. Localized viscous shearing in the reference model leads to strain rates that are 3–4 orders of magnitudes higher in the weak zones than in the garnet-rich strong domains (Fig. 4c), which further increases the effective viscosity contrast to 5–6 orders of magnitude. Strain rate in the weak zone exceeds 10^–14^ s^−1^, which is more than an order of magnitude higher than the imposed shearing rate. Such localization of deformation in the weak zone network leads to very low strain rate in the strong domains (around 10^–17^ 1/s, Fig. 4c), which in turn produces low overall differential stress levels in the reference model (Fig. 4d,e). Average differential stress in the plotted domain (the area of Fig. 4d) is 13 MPa at 50 kyr and 23 MPa at 1 Myr (Fig. 4e). The latter is considered a good approximation of the long-term value due to the extremely slow accumulation of stress at this point in time (the rate of stress buildup here is 1–2 MPa/Myr).

When considering a geometry where weak zones are scarcer and occupy less space (17% of the model area in Model B, wrt. to 27% in the reference model, Fig. 4a,b), differential stress magnitudes are significantly higher (Fig. 4g,h). Strain rate in the strong domains remains 2–3 orders of magnitude lower than in the weak zones, however, it is generally higher than in the reference model (Fig. 4c,f) due to the scarcer weak zone network. This increases long-term average differential stress in Model B by a factor of ~ 2 (Fig. 4h).

**Fig. 5:**
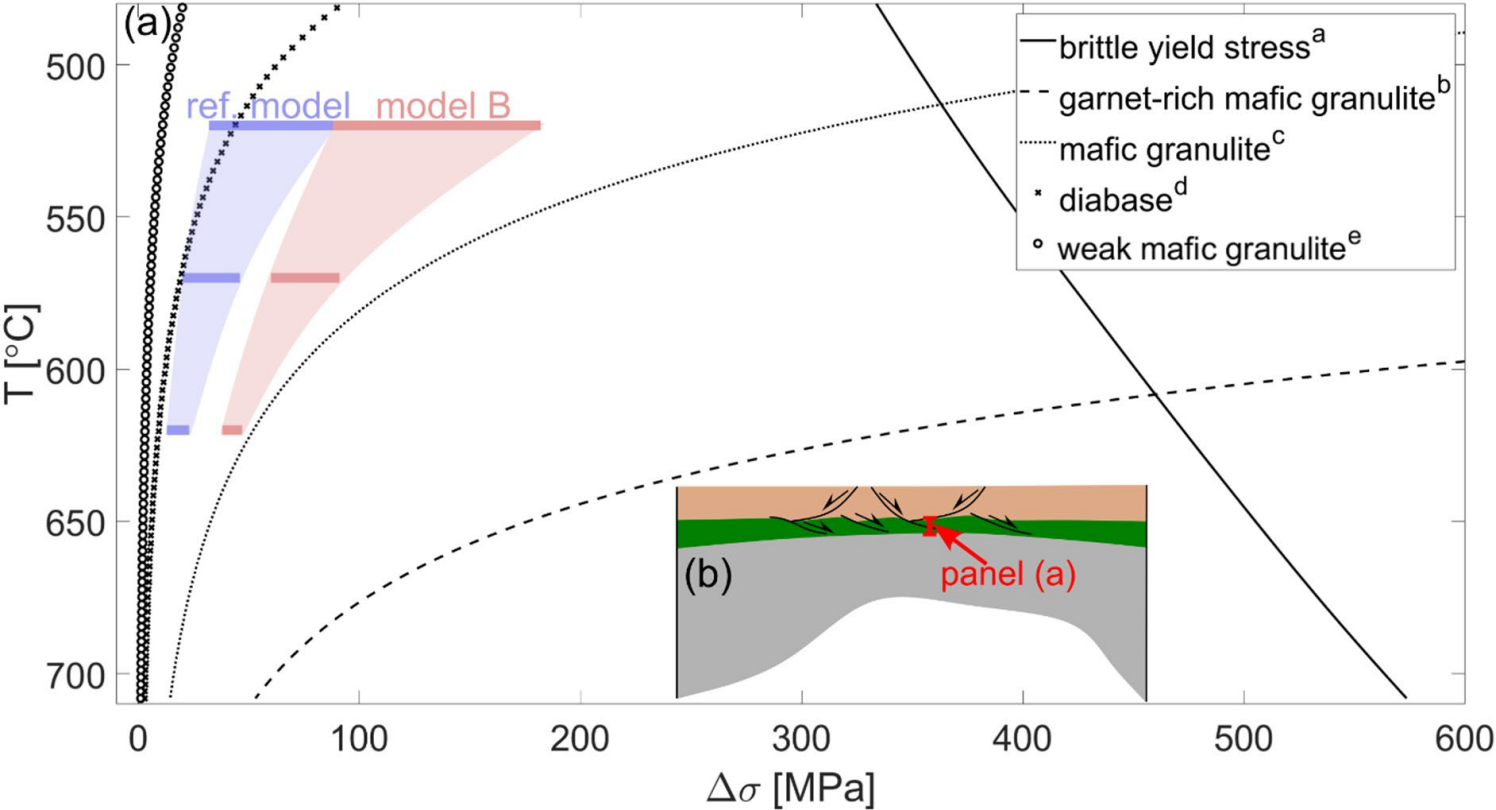
**a** Comparison of differential stress ($\Delta$**σ**) magnitudes recorded in the numerical models and the flow stress of different rock types, calculated for present-day lower crust temperatures (T) of the BBHVF area in the Pannonian Basin (schematic section of panel **b**). Curves labelled a, c, d, and e in the figure are based on^27,28,31,70^, respectively (curve ^e^ is the flow law used for weak zones in the numerical models). Curve ^b^ is based on a 50–50% mixture of garnet-free mafic granulite^32^ and garnet^33^, using the aggregate law of^34^; the same flow law is used for strong domains in the numerical models. Strain rate is set to 10^–15^ s^−1^ for flow law calculations, equaling the imposed strain rate in the numerical models (see Fig. S3 for results when the strain rate is set to 10^–16^ s^−1^ for both numerical model and flow law calculations). The same temperature profile and brittle parameters are used as in Fig. 1d. Blue and red lines are results of the reference model and model B for T = 520, 570, and 620 °C, respectively. The left limit of the blue/red lines is average differential stress in the model domain at 50 kyr, while the right limit is at 1 Myr. Average differential stress is tracked within the plotted domain of Fig. 4c,d,f,g, to avoid any boundary effects at the edges of the model. Transparent blue/red areas connecting the model results represent likely T- $\Delta$**σ** values for cases that were not modelled.

We consider the average long-term differential stress magnitude in the modelled heterogenous lower crust domains as the proxy for the bulk rheological behavior (strength or flow stress) of the system (Fig. S2g shows for a homogenous material that average long-term differential stress in our numerical model is equivalent to the analytical flow stress of the same material). As average value, we use the arithmetic mean of the differential stress magnitude. This allows us to compare the stress levels of modelled heterogenous systems with flow laws of different lower crust materials typically used in large-scale geodynamic models (curves ^c^ and ^d^ on Fig. 5a) as well as with flow laws that we use for defining strong domains and weak zones in the numerical model (curves ^b^ and ^e^ on Fig. 5a). This comparison shows that despite the strong lithology occupying 73% of the reference model, the average differential stress is much closer to the flow stress of the weak zone rheology (curve ^e^ on Fig. 5a) than that of the strong domain rheology (curve ^b^ on Fig. 5a). The average differential stress in the reference model is very close to the flow stress of Maryland diabase^27^, a flow law that is often used for large-scale geodynamic modelling, also for the Pannonian Basin^47^. Besides the reference model that is fixed at 620 ^o^ C, we also tested the same setup (Fig. 4a) for 570 and 520 °C (Fig. 5a). Differential stress magnitudes in the three models with different temperatures outline an approximate trend of a flow law that describes the strength of the modelled heterogenous lower crust domain. Considering stress magnitudes at 50 kyr and 1 Myr (the left and right limits of blue/red lines on Fig. 5a) results in a range of possible medium-to long-term stress magnitudes for the heterogenous lower crust. Models with lower temperatures show more significant stress buildup between 50 kyr and 1 Myr (Fig. 5a), which can be explained by the increasingly elastic mode of deformation (higher Deborah numbers due to increasing viscosity at lower temperatures^77,78^).

In cases where weak zones are scarcer and/or less evenly distributed, long-term average differential stress is significantly higher. In such a case, the flow stress of a moderately strong (but still garnet-free) mafic granulite flow law^28,32^ fits better the modelled stress levels (Fig. 5). Furthermore, in cases where weak zones are isolated/disrupted, fast shearing in the weak zones is locally transferred through strong domains as well (Fig. S2a,b), giving rise to extremely high differential stresses that locally exceed the brittle yield stress (Fig. S2e) and would possibly induce earthquakes in nature.

Keeping the reference model geometry (Fig. 4a) but changing the thickness of each individual weak zone by ca. 30% reveals a moderate influence of weak zone thickness on the stress levels: thinner weak zones lead to higher differential stress magnitudes (Fig. S4). The effect of weak zone thickness (as long as the weak zones are well resolved in the model) is not as important as continuity or spacing. We also tested the effect of strength/viscosity contrast between the weak and strong domains by modifying the flow law of the weak zones. This shows that further increasing the contrast has insignificant impact, while decreasing it leads to less efficient strain localization in the weak zones, bringing differential stress magnitudes closer to the strong domains’ flow law (Fig. S5).

## Discussion

Results show that continuous, densely spaced weak zone networks surrounding strong lithologies significantly decrease overall stress magnitudes, even if the weak material only constitutes a relatively small portion (20–25%) of the rock volume. Viscous shearing is highly localized in the weak zones (Fig. 4), resulting in average differential stress decrease by a factor of 10–20, compared to a case without weak zones (Fig. 5a, S2). This mechanical behaviour resembles the concept of ‘melt connectivity transition’ in partially melted rocks, where a small amount of melt, if interconnected, dramatically decreases overall rock strength^79^. This means that lower crust domains that mainly consist of strong lithologies, may still behave as weak layers in the lithosphere, when localized metamorphic reactions create long-term weak zones. Such weakening could explain the apparent contradiction between the weak large-scale mechanical behavior and observed strong lithological composition of the AlCaPa lower crust in the Pannonian Basin. Lower crust xenolith samples show that weak zones formed due to garnet breakdown and elevated water content, surrounding the strong and dry garnet-rich domains (Fig. 2). This network of weak zones may facilitate overall weak behaviour; average differential stress magnitude in the lower crust could be close to the flow stress of relatively weak materials, such as diabase^27^ (Fig. 5a). The phenomenon of overall lower crust weakening due to localized retrograde reactions hence may justify the choice of relatively weak flow laws for the large-scale modelling of the Pannonian Basin system, both for rifting^47^ and inversion^53^ processes. Results also indicate that scarce or discontinuous weak zone networks result in stress magnitudes that are closer to the flow stress of moderately strong (but still garnet-free) mafic granulite flow laws^28,32^ (Fig. 5, S2g). In such cases the lower crust would likely behave as a relatively strong layer in the lithosphere (Figs. 1b,d, 5), leading to significantly different patterns of large-scale extension, contraction, and possibly seismicity, than observed in the Pannonian Basin. We note that wide rift patterns matching the geometry of the Pannonian Basin have been reproduced also by using initially strong lower crust rheology, however, this requires the convective thinning of the lithospheric mantle prior to the main phase of extension^80^, indicating that further weakening mechanisms are needed to explain the natural case. Based on these considerations, we argue that the AlCaPa lower crust in the Pannonian Basin contains continuous and relatively densely spaced weak zone networks (e.g. Fig. 4a) that surround the strong, dry, garnet-rich domains, accommodating most of the long-term deformation and in turn enabling large-scale weak behaviour. We emphasize that the inherent uncertainty of extrapolating from xenolith samples and analogous exposed lower crust bodies leaves room for alternative/additional interpretations for the Pannonian case. We also note that the lower crustal rheology of the entire Pannonian region cannot be explained by AlCaPa-derived observations and models alone, as the southern part of the basin is underlain by the Europe-derived Tisza-Dacia nappe stack (Fig. 2a) with an unconstrained lower crustal composition. While unconstrained, the present-day structural and thermo-mechanical continuity of the Tisza-Dacia unit with the AlCaPa unit^81,82^ and its even more significant crustal thinning^37^ implies that the Tisza-Dacia lower crust is at least similarly weak to the AlCaPa lower crust.

Overall weakening due to localized metamorphic reactions and fluid/melt migration may be a key element in understanding the mechanical behaviour of ductile lithosphere layers worldwide. Mantle-or locally-derived fluid/melt migration through the lithosphere inevitably creates compositional and rheological contrasts^73,83^, producing highly heterogenous systems with a large-scale mechanical behavior that may be very different from that of the dominant lithology, as demonstrated by our results. We emphasize that besides “permanent” or long-term weakening—related to compositional changes that we consider in our model—transient weakening due to reactions and melt/fluid migration processes^84–86^ may also be significant. These effects may further increase viscosity contrasts between strong and weak domains, producing an even higher degree of strain localization in the weak zones, and even lower average stress magnitudes.

Continuous and densely spaced weak zone networks hosted by a strong lithology yield very limited stress buildup: long-term differential stress stays hundreds of MPa below the brittle yield stress for relatively slow strain rates of 10^–15^ s^−1^ or 10^–16^ s^−1^ (Figs. 5, S3, respectively). This agrees well with models investigating stress buildup in strong blocks, which demonstrated that much higher strain rates are required for reaching the failure criterion^87^. However, our results show that even slow strain rates may induce high enough stresses for brittle failure when weak zones are discontinuous (Fig. S2g): in model C, maximum differential stress level reaches the brittle yield at 70 kyr (Fig. S2), meaning that such systems could potentially produce lower crustal earthquakes, albeit probably with extremely long return periods.

## Methods

### Micro fourier-transform infrared spectrometry (micro-FTIR)

Micro-FTIR measurements have been undertaken at the Hungarian Academy of Sciences using a Varian 7000 Spectrometer attached to an UMA600 microscope. KBr beam splitters and MCT detectors were deployed with a Globar light source. Rectangular spot size varied between 50 * 50 and 100 * 100 microns depending on the size of the target mineral. Nominal spectral resolution of 4 cm^−1^ was chosen with at least 64 scans between usually between 400 and 4000 cm^−1^ for each measurement.

Spectra were processed by the OPUS^®^ software. For the background correction the concave rubber band correction including at least 2 iterations and 64 fitting points was applied, but the number of iterations may have been more (up to 4) for spectra with uneven background. Integration was completed using the Integration tool with the B method which includes only the area above the line connecting the intersections of the lower and upper limits of integration with the spectra. Usually, uniform limits of integration were applied for each mineral.

The method of^88,89^ makes it possible to determine the concentration of ‘water’ from a number (n > 5) of unoriented anisotropic crystals with good accuracy. This method could only be applied for strongly anisotropic minerals (e.g., olivine, calcite), if the maximum linear unpolarized absorbance is less than 0.15, which is met in our study. Total polarised absorbance (A_tot_) is estimated as three times the average unpolarized absorbance. The estimation is more accurate if as many as possible unoriented grains are considered for the average integrated unpolarized absorbance covering a wide range of indicatrix orientations with respect to the incoming radiation.

The A_tot_ is then converted to absolute concentration of ‘water’ (ppm wt%) using the integrated molar extinction coefficients (ε) and densities (ρ) for different NAMs of the studies lower crustal granulite xenoliths according to Eq. (1):

1 $$c \left( {ppm \,wt.\% \,H_{2} O} \right) = \frac{{M_{A} \cdot A_{{\frac{tot}{{cm}}}} }}{\rho \cdot \in } \cdot 10^{6}$$

where *M*_*A*_ is the molar mass of H_2_O (18.02 g/mol); *A*_*tot/cm*_ is the total integrated absorbance normalised to 1 cm thickness, $\in$ is in L·mol^−1^H_2_O cm^−2^), *ρ* is in g/L. Densities for garnet, pyroxenes and feldspar were estimated based on their respective average geochemical compositions according to^69^.

There are typical uncertainties involved in the absolute concentration of water including those in the thickness of thin sections (± 3%), total integrated polarised absorbances (± 10%), estimated densities (± 5%) and calibration factors (± 10%). Based on prior experience^88^, this usually translates to an overall precision of ~ 15% in the absolute concentration of ‘water’ but should not exceed 30% even in the worst case.

### Numerical model

To model visco-elastic deformation, we numerically solve the momentum and continuity equations using 2D Cartesian (*x*,*y*) coordinates, assuming isothermal conditions. We employ finite difference discretization on a staggered grid (e.g^90^.) and an accelerated pseudo-transient solving strategy (e.g^77,91^.). Tolerance value test for pseudo-transient iteration loops and a numerical resolution test serves as validation for model accuracy and is provided in Fig. S6.

By neglecting gravity and inertial terms, the momentum equations take the form of

2 $$- \frac{\partial P}{{\partial x}} + \frac{{\partial {\boldsymbol{\tau}}_{xx} }}{\partial x} + \frac{{\partial {\boldsymbol{\tau}}_{xy} }}{\partial y} = 0$$

3 $$- \frac{\partial P}{{\partial y}} + \frac{{\partial {\boldsymbol{\tau}}_{yy} }}{\partial y} + \frac{{\partial {\boldsymbol{\tau}}_{yx} }}{\partial x} = 0$$

where ${\boldsymbol{\tau}}$ denotes deviatoric stress tensor components and *P* is the pressure or mean stress. The continuity equation for incompressible flow is expressed as

4 $$\frac{{\partial {\boldsymbol{v}}_{x} }}{\partial x} + \frac{{\partial {\boldsymbol{v}}_{y} }}{\partial y} = 0$$

where ***v***denotes the two velocity components. To compute deviatoric strain rates and stresses, we implement a Maxwell visco-elastic model (e.g^92^.,):

5 $$\dot{\user2{\varepsilon }}_{ij} = \dot{\user2{\varepsilon }}_{ij}^{v} + \dot{\user2{\varepsilon }}_{ij}^{e} = \frac{{{\boldsymbol{\tau}}_{ij} }}{2\eta } + \frac{{\dot{\user2{\tau }}_{ij} }}{2G}$$

where, ${\dot{{\boldsymbol{\varepsilon}}}}_{ij}^{v}$ is the viscous and ${\dot{{\boldsymbol{\varepsilon}}}}_{ij}^{e}$ is the elastic strain rate component, ${\dot{{\boldsymbol{\tau}}}}_{ij}$ is the time derivative of the deviatoric stress, $\eta$ is the effective viscosity, and *G* is the shear modulus (57 and 70 GPa for garnet-free granulite weak zones and garnet-rich domains, respectively, based on plagioclase^93^, clinopyroxene^94^ and garnet^95^ data). Deviatoric stresses are computed as

6 $${\boldsymbol{\tau}}_{ij} = 2\eta_{VE} \dot{\user2{E}}_{ij}^{\prime }$$

where ${\eta }_{VE}$ is defined as a visco-elastic modulus, which is computed using effective viscosity ($\eta$), shear modulus (*G*), and the time step ($\Delta t$) used for discretization:

7 $$\eta_{VE} = \left( {\frac{1}{\eta } + \frac{1}{G\Delta t}} \right)^{ - 1}$$

${\dot{{\boldsymbol{E}}}}_{ij}{\prime}$ in Eq. (6) represents effective visco-elastic strain rates defined as

8 $$\dot{\user2{E}}_{ij}{\prime} = \dot{\user2{\varepsilon }}_{ij} + \frac{{{\boldsymbol{\tau}}_{ij}^{o} }}{2G\Delta t}$$

where ${{\boldsymbol{\tau}}}_{ij}^{o}$ represents deviatoric stresses computed in the previous time step^92^.

The effective viscosity is a function of material properties, temperature (*T*) and strain rate, and is expressed using geometrical correction factors (e.g^13^.,):

9 $$\eta = \frac{{2^{{\frac{1 - n}{n}}} }}{{3^{{\frac{1 + n}{{2n}}}} }}A^{{\frac{ - 1}{n}}} \varepsilon_{II}^{{\frac{1}{n} - 1}} {\mathrm{exp}}\left( \frac{Q}{nRT} \right)$$

where material properties *A*, *n*, *Q* are derived from laboratory experiments and are different for weak zones and strong domains in the model (*A* = 6.31 * 10^–22^ Pa^−n^.s^−1^, *n* = 3.2, *Q* = 244 kJ/mol for moderately wet, weak, garnet-free mafic granulite^76^; *A* = 8.73*10^–14^ Pa^−n^.s^−1^, *n* = 3, *Q* = 467 kJ/mol for stronger garnet-rich mafic granulite domains^32–34^). ${\dot{\varepsilon }}_{II}$ is the second invariant (magnitude) of the deviatoric strain rate tensor, *R* is Bolzman’s gas constant.

Differential stress is used to quantify the strength of the modelled lower crust domain, allowing comparison with the flow stress of different materials (Fig. 5). Differential stress ($\Delta{\boldsymbol{\sigma}}$) is defined as (e.g^96^.,)

10 $$\Delta {\boldsymbol{\sigma}} = 2\tau_{max}$$

where ${\tau }_{max}$ is the effective or maximum shear stress (equivalent to the second invariant of the deviatoric stress tensor, ${\tau }_{II}$), expressed using total stress tensor (${\boldsymbol{\sigma}}$) components as:

11 $$\tau_{max} = \tau_{II} = \sqrt {\left( {\frac{{\left( {{\boldsymbol{\sigma}}_{xx} } \right) - \left( {{\boldsymbol{\sigma}}_{yy} } \right)}}{2}} \right)^{2} + {\boldsymbol{\sigma}}_{xy}^{2} }$$

## Supplementary Information

Supplementary Information.

## Data Availability

The authors declare that all data supporting the findings of this study are available within the paper and its supplementary information file.

## Code availability

The numerical code of the reference model presented in this study is available in^97^.
