# Supplementary material for: Large-scale rheological and tectonic effects of localized garnet breakdown: a case from the Pannonian Basin
Source: Sci Rep. 2026 Apr 18;16:22730. doi: 10.1038/s41598-026-43790-4 (PMC13385591; doi:10.1038/s41598-026-43790-4)
Supplement: Supplementary file 1 — Supplementary Information. [file 41598_2026_43790_MOESM1_ESM.pdf]

SUPPLEMENTARY Information to

Large-scale rheological and tectonic effects of localized garnet breakdown: a case from the Pannonian Basin

Kristóf Porkoláb[1], Kálmán Török[2, 1], Tamás Sránitz[1], István Kovács[1], Eszter Békési[1], Márta Berkesi[1]

[1]MTA-EPSS FluidsByDepth Lendület (Momentum) Research Group, HUN-REN Institute of Earth Physics and Space Science, 9400 Sopron, Hungary

[2]Supervisory Authority for Regulatory Affairs, Budapest, Hungary

		fdp		grt		opx		cpx		ru		qtz		sil	
Sample	t (µm)	ppm	na	ppm	na	ppm	na	ppm	na	ppm	na	ppm	na	ppm	na
SAB36	156	-		**bld**	*16*	-		-		**155**	*8*	**90**	*15*	**86**	*10*
SAB31	93	**34**	*4*	**bld**	*17*	-		-		-		**64**	*10*	**76**	*11*
SAB141	107	**61**	*10**	**bld**	*9*	-		-		**34**	*7*	-		**97**	*15*
Mi71	169	**66**	*10*	**bld**	*11*	-		-		**101**	*4*	-		-	
SAB38wall	100	**bld**	*7*	-		-		**53**	*11*	-		-		-	
SAB38vein	100	**bld**	*10*	-		-		**71**	*10*	-		**22**	*2*	-	
Mi26	107	**97**	*32**	**bld**	*2*	-		**92**	*21*	-		-		-	
SAB8	176	**47**	*11*	-		**58**	*7*	**272**	*15*	**698**	*4*	-		-	
Mi115	185	**81**	*9**	**495**	*7**	-		**251**	*12*	-		-		-	
Mi82	142	**23**	*7*	**113**	*2**	-		**151**	*6*	-		-		-	
Mi10	153	**43**	*12*	**264**	*27**	**55**	*8*	-		**41**	*2*	-		-	
Mi19	145	**26**	*15**	**bld**	*8*	**57**	*12*	-		-		-		-	
SAB14	100	-		**bld**	*12*	-		**381**	*13*	-		-		-	
SAB32	156	**108**	*13**	**21**	*10*	-		**295**	*12*	-		-		-	
Mi21	142	**63**	*10*	**bld**	*9*	-		**157**	*10*	-		-		-	
Mi77	82	**31**	*17*	**bld**	*27*	-		-		-		-		-	

t = thickness; na = number of analysis; ppm = in molecular water equivalent ppm wt.%; fdp = feldspar; grt = garnet; opx = ortopyroxene; cpx = clinopyroxene; ru = rutile; qrtz = quartz; sil =sillimanite; bld = below the limit of detection; * = high wavenumber bands at ~3695 and 3570 cm^{-1} indicate the presence of hydrous alteration products, thus concentrations are likely overestimated

Table S1. H_2O contents of nominally anhydrous minerals in the studied granulite xeoliths from the Bakony-Balaton Highland. The xenolith samples are linked to two volcanic edifices: 1) Mt. Sabar (lat: 46.848 , lon: 17.530), located at the village of Káptalantóti. Sample code SAB indicates origin from this location. 2) Mt. Kopasz (lat: 46.872, lon: 17.545), located at the village of Mindszentkálla. Sample code Mi indicates origin from this location.

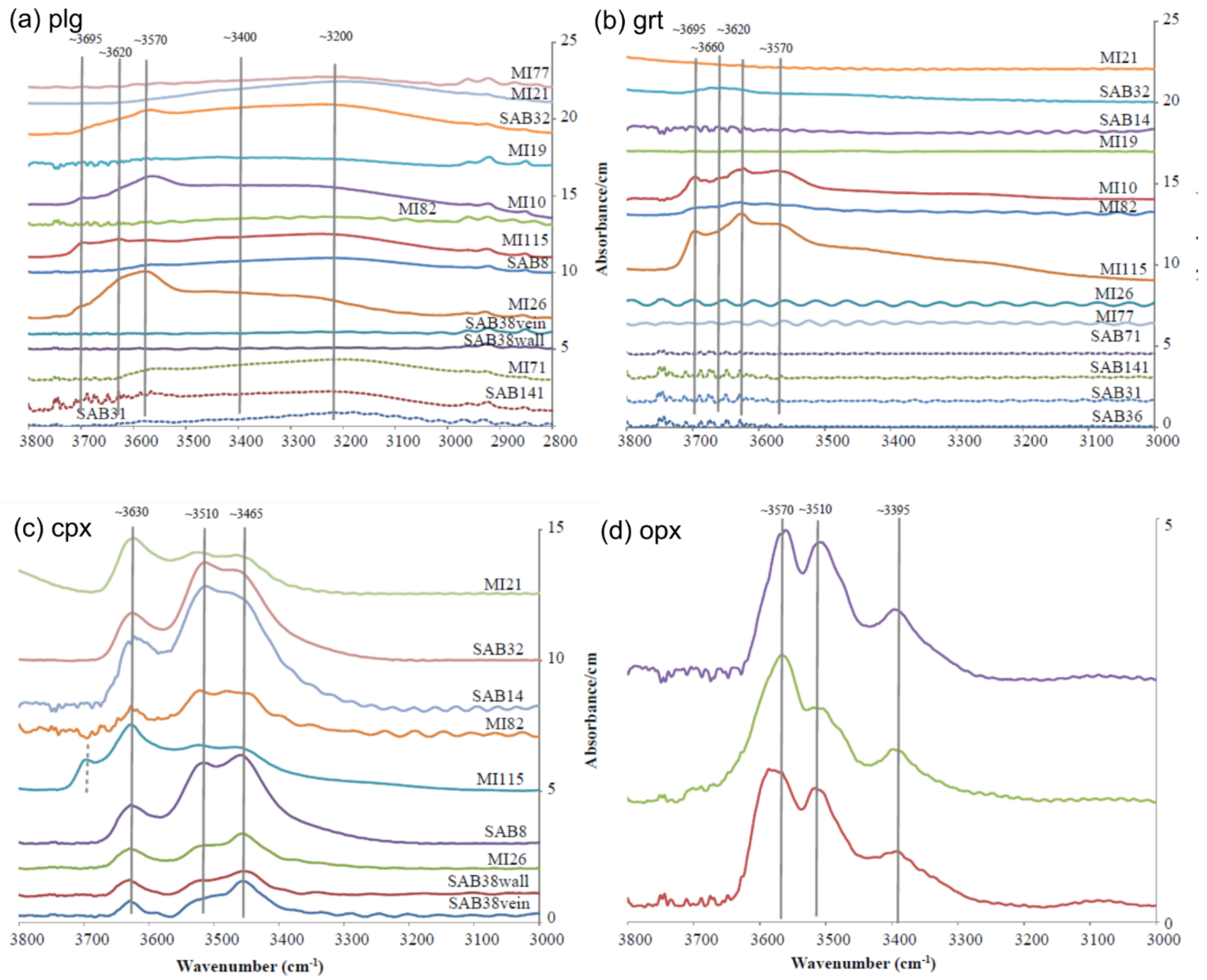

Figure S1. Micro-FTIR spectra of plagioclase (a), garnet (b), clinopyroxene (c), and orthopyroxene (d).

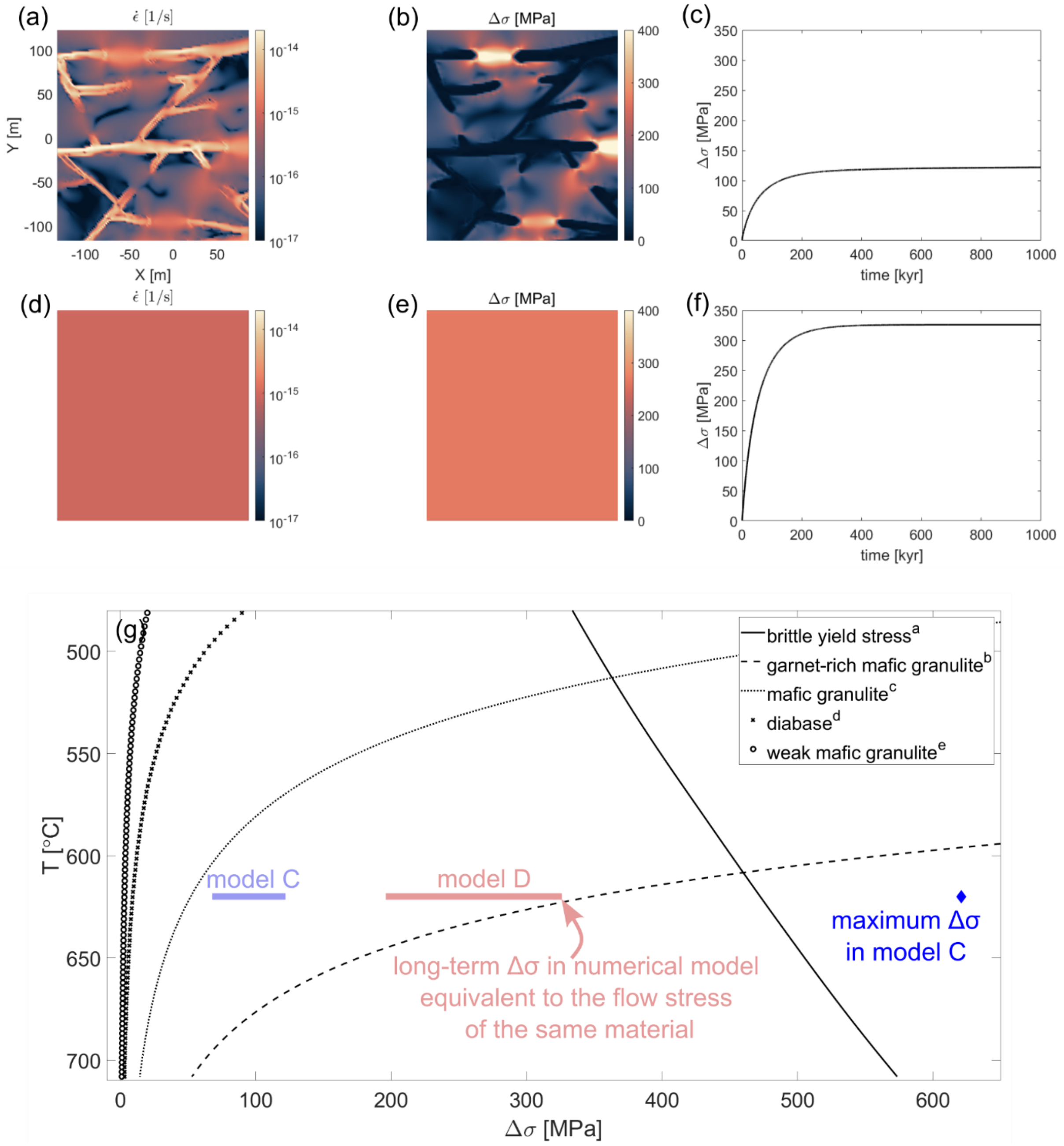

Figure S2. (a) Strain rate ($\dot{\varepsilon}$) in model C (the reference model geometry displayed by Figure 4a in the main text is modified by disrupting the weak zones) after 70 kyr. **(b)** Average differential stress ($\Delta\sigma$) in model C after 70 kyr. **(c)** Time evolution of average differential stress in model C. **(d)** Strain rate in model D (homogenous case consisting entirely of the strong lithology, garnet-rich mafic granulite) after 70 kyr. **(e)** Average differential stress in model D after 70 kyr. **(f)** Time evolution of average differential stress in model D. **(g)** Comparison of differential stress magnitudes recorded in the numerical models and the flow stress of different rock types, calculated for present-day lower crust temperatures (T) of the BBHVF area in the Pannonian Basin (schematic section of panel b). Flow/Yield stress curves

are the same as on Figure 5 in the main text. Strain rate is set to 10^{-15} s^{-1} for flow law calculations, equaling the imposed strain rate in the numerical models. The same temperature profile and brittle parameters are used as in Figure 1d. Blue and red lines are results of model C and model D for T = 620 °C, respectively. The left limit of the blue/red lines is average differential stress in the model domain at 50 kyr, while the right limit is at 1 Myr. Model D consists entirely of the same material that describes flow law [b], hence, the coinciding right limit of the differential stress range with this flow law confirms that long-term differential stress in our model corresponds to the flow stress of the corresponding material. Average differential stress is tracked within the plotted domain of Figure S2 a, b, d, and e, to avoid any boundary effects at the edges of the model. Blue diamond represents the maximum differential stress reached after 1 Myr in model C, showing that extremely high local stresses in this setup exceed the brittle yield stress.

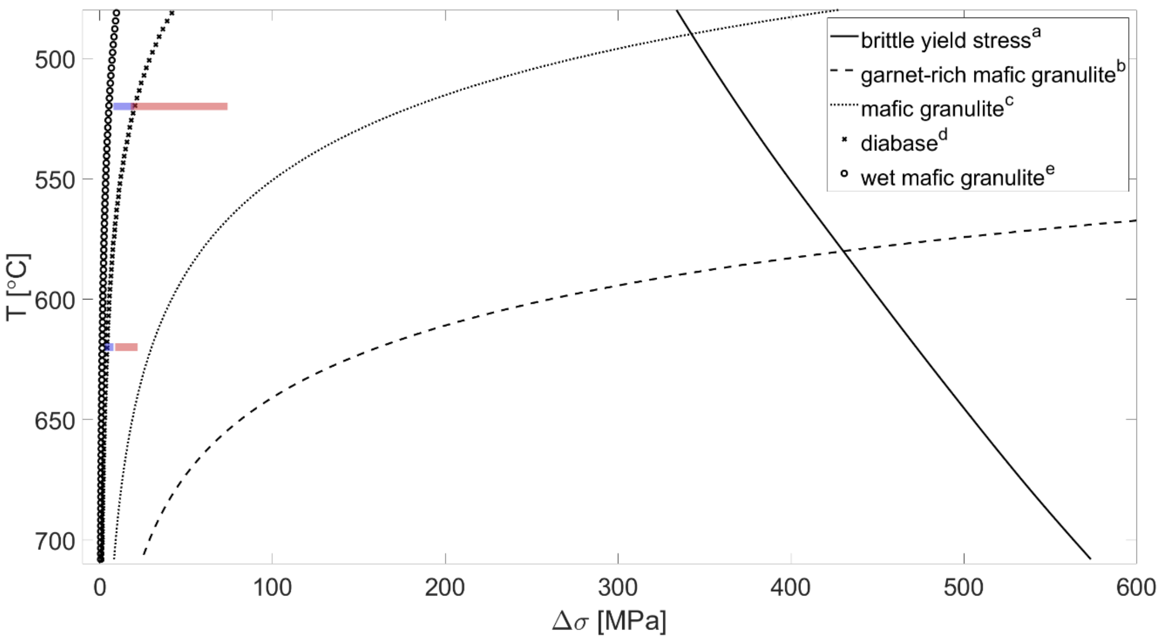

Figure S3. Differential stress magnitudes recorded in numerical models that share the same geometry as the reference model (blue lines) and model B (red lines) but deform an order of magnitude slower (10^{-16} s^{-1}). The left limit of the blue/red lines is average differential stress in the model domain at 50 kyr, while the right limit is at 1 Myr. Flow stress of different rock types are calculated for present-day lower crust temperatures (T) of the BBHVF area in the Pannonian Basin (schematic section of panel b). Flow/Yield stress curves are based on the same parameters as on Figure 5 in the main text. Strain rate is also set to 10^{-16} s^{-1} for flow law calculations. The same temperature profile and brittle parameters are used as in Figure 1d.

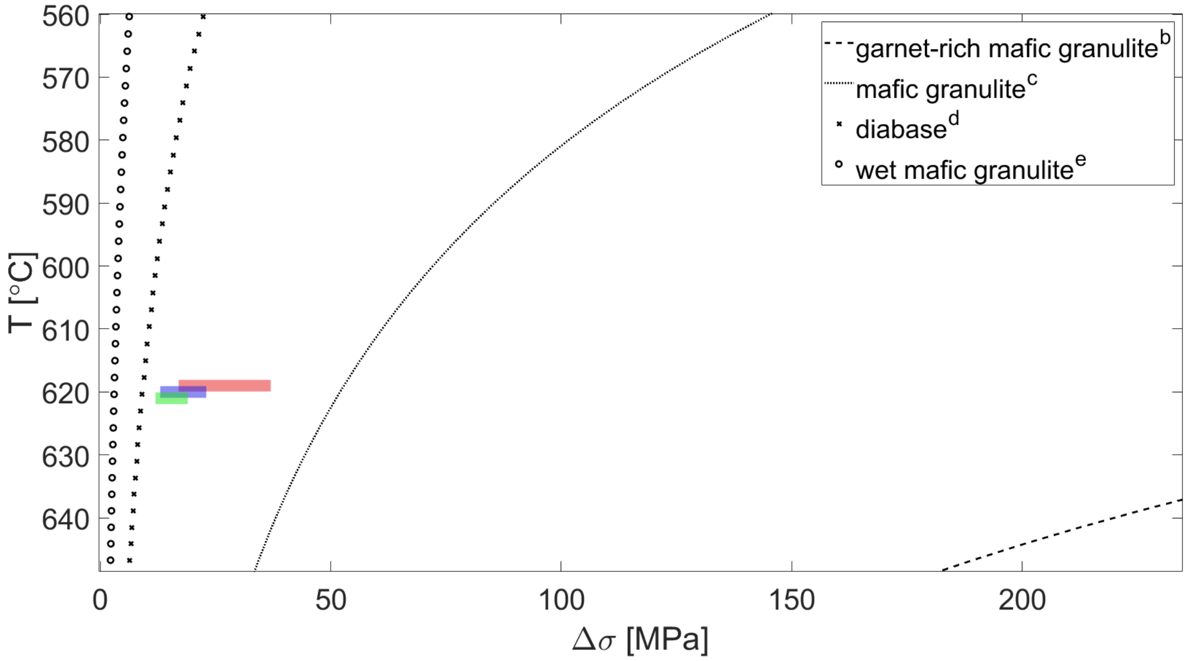

Figure S4. Differential stress magnitudes recorded in numerical models with identical weak zone network geometry to the reference model but variable weak zone thickness values. Blue line is the reference model (27% of model domain covered by weak zones), green line is a model with ca. 30% thicker weak zones (32% of model domain covered by weak zones), red line is a model with ca. 30% thinner weak zones (21% of model domain covered by weak zones). All models are fixed at 620 °C, the slight vertical shift between the lines serves visual clarity only. The left limit of the colored lines is average differential stress in the model domain at 50 kyr, while the right limit is at 1 Myr. Flow stress of different rock types are calculated for present-day lower crust temperatures (T) of the BBHVF area in the Pannonian Basin (schematic section of panel b). Flow/Yield stress curves are based on the same parameters as on Figure 5 in the main text. The same temperature profile and brittle parameters are used as in Figure 1d.

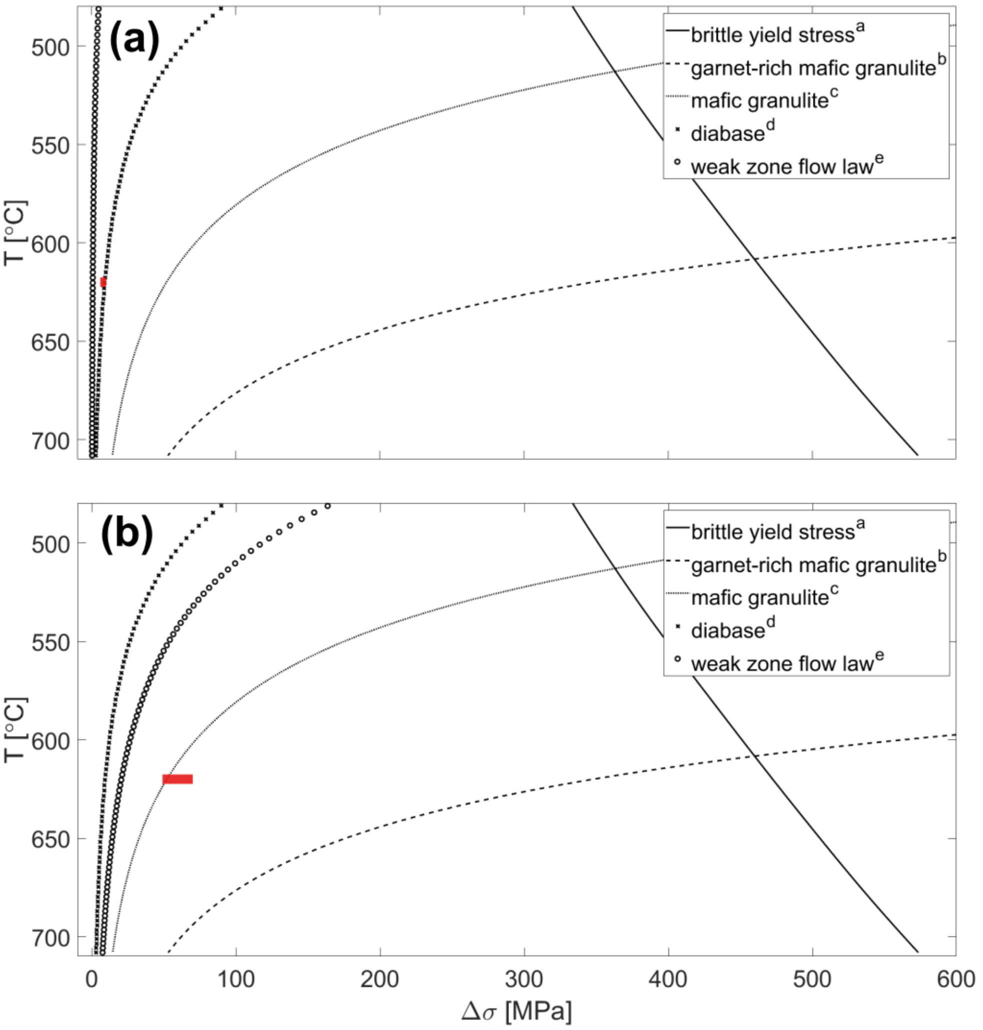

Figure S5. Differential stress magnitudes recorded in numerical models with identical weak zone network geometry to the reference model but variable weak zone strength (achieved by modifying the weak zone flow law). **(a)** The case of weakened weak zones (see flow law [e]), which increases the viscosity contrast between weak and strong domains. Model behavior is very similar to the reference case, with slightly lower average stress levels. **(b)** The case of strengthened weak zones (see flow law [e]), which decreases viscosity contrast. Decreasing level of strain localization in the weak zones results in higher overall stress levels. This indicates that high strength contrast makes the overall weakening much more significant. For both panels: left limit of the red lines is average differential stress in the model domain at 50 kyr, while the right limit is at 1 Myr. Flow stress of different rock types are calculated for present-day lower crust temperatures (T) of the BBHVF area in the Pannonian Basin (schematic section of panel b). Flow/Yield stress curves are based on the same parameters as on Figure 5 in the main text. The same temperature profile and brittle parameters are used as in Figure 1d.

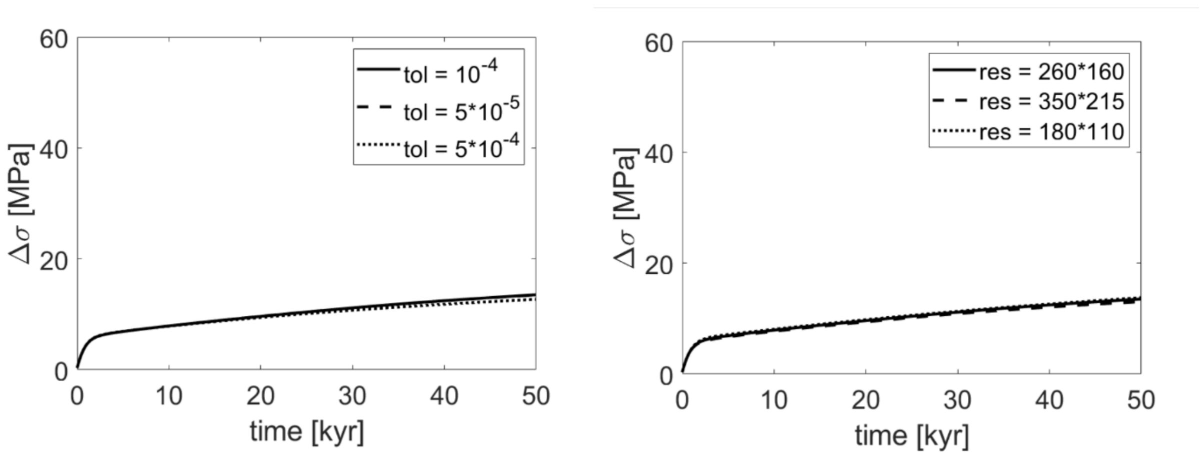

Figure S6. Numerical tolerance (left) and resolution (right) tests of the reference model. The reference model has a tolerance value of 10^{-4} and a resolution of 260*160. Smaller tolerance value means that the pseudo-transient iteration loop, where the equation system is solved, is required to iterate longer to further reduce differential equation residuals. The test shows that decreasing this tolerance value does not alter stress level predictions, which is the main result of the models. Increasing the tolerance value to $5*10^{-4}$ produces marginally different stress predictions, which we can consider less precise compared to the reference model. Increasing or slightly decreasing the resolution of the model also does not lead to significant changes, only a marginal difference can be detected in stress predictions.
